# NADPH Oxidase (Rboh) Activity is Up Regulated during Sweet Pepper (*Capsicum annuum* L.) Fruit Ripening

**DOI:** 10.3390/antiox8010009

**Published:** 2019-01-01

**Authors:** Ángela Chu-Puga, Salvador González-Gordo, Marta Rodríguez-Ruiz, José M. Palma, Francisco J. Corpas

**Affiliations:** Group Antioxidant, Free Radical and Nitric Oxide in Biotechnology, Food and Agriculture, Department of Biochemistry, Cell and Molecular Biology of Plants, Estación Experimental del Zaidín, CSIC, C/Profesor Albareda 1, E-18008 Granada, Spain; angelachupuga@gmail.com (Á.C.-P.); salvador.gonzalez@eez.csic.es (S.G.-G.); martarodriguezruiz@usp.br (M.R.-R.); josemanuel.palma@eez.csic.es (J.M.P.)

**Keywords:** NADPH oxidase, nitric oxide, nitration, pepper fruit, peroxynitrite, respiratory burst oxidase homolog (Rboh), *S*-nitrosation, ripening, Tyr-nitration

## Abstract

In plants, NADPH oxidase (NOX) is also known as a respiratory burst oxidase homolog (Rboh). This highly important enzyme, one of the main enzymatic sources of superoxide radicals (O_2_•^−^), is involved in the metabolism of reactive oxygen and nitrogen species (ROS and RNS), which is active in the non-climacteric pepper (*Capsicum annuum* L.) fruit. We used sweet pepper fruits at two ripening stages (green and red) to biochemically analyze the O_2_•^−^-generating Rboh activity and the number of isozymes during this physiological process. Malondialdehyde (MDA) content, an oxidative stress marker, was also assayed as an index of lipid peroxidation. In red fruits, MDA was observed to increase 2-fold accompanied by a 5.3-fold increase in total Rboh activity. Using in-gel assays of Rboh activity, we identified a total of seven CaRboh isozymes (I–VII) which were differentially modulated during ripening. CaRboh-III and CaRboh-I were the most prominent isozymes in green and red fruits, respectively. An in vitro assay showed that CaRboh activity is inhibited in the presence of nitric oxide (NO) donors, peroxynitrite (ONOO^−^) and glutathione (GSH), suggesting that CaRboh can undergo *S*-nitrosation, Tyr-nitration, and glutathionylation, respectively. In summary, this study provides a basic biochemical characterization of CaRboh activity in pepper fruits and indicates that this O_2_•^−^-generating Rboh is involved in nitro-oxidative stress associated with sweet pepper fruit ripening.

## 1. Introduction

NADPH oxidase (NOX) is considered to be the most important enzyme responsible for superoxide radicals (O_2_•^−^) generation in mammalian cells. In humans, seven genes encoding NOX isozymes, involved in a wide range of cellular processes including apoptosis, host defense, cellular signal transduction, oxygen sensing, and angiogenesis have been identified [1]. In plants, the NOX enzyme is referred to as a respiratory burst oxidase homolog (Rboh). It is composed of six conserved transmembrane domains, the C-terminal harboring FAD and NADPH hydrophilic domains, two heme groups, and two N-terminal Ca^2+^-binding EF-hand motifs indicating that Rboh activity is regulated by Ca^2+^. Although the Rboh gene is ubiquitously expressed, the distribution and abundance of its different isozymes are cell- and tissue-specific. This suggests that each Rboh isozyme is involved in distinct physiological and stress functions, including seed germination, root hair formation, lignification, stomatal closure, senescence, systemic signaling, pollen tube growth, as well as abiotic and biotic stress [2,3,4,5,6,7,8,9,10], and in establishing symbiotic associations with *Rhizobium* [11,12]. 

Unlike tomato (*Solanum lycopersicum* L.), pepper (*Capsicum annuum* L.), which also belongs to the Solanaceae family, is a non-climacteric fruit due to its ethylene-independent ripening [13]. The numerous pepper fruit varieties differ in shape, color, and capsaicin content, but are all characterized by high vitamin C content [14]. Fruit ripening is a genetically programmed physiological process involving many phenotypical modifications that reflect the profound biochemical and molecular changes taking place during this developmental stage. In the case of sweet peppers, previous studies have indicated that different subcellular compartments, including peroxisomes, mitochondria, and plastids, are involved in the active metabolism of reactive oxygen species (ROS) and reactive nitrogen species (RNS) [15,16,17,18,19]. Thus, there is significant information indicating that NADPH-generating enzymes and antioxidant systems are modulated to different degrees [15,16,20,21,22]. Proteomic analyses have also shown that sweet pepper fruit ripening is accompanied by an increase in protein nitration, a NO-derived post-translational modification which triggers the inhibition of the antioxidant catalase, one of the most abundant proteins identified by proteomic approaches [23]. Ascorbate biosynthesis and *S*-nitrosothiols are also differentially modulated by NO [24,25]. However, to our knowledge, no information exists on how O_2_•^−^-generating Rboh activity is modulated during pepper fruit ripening and on the number of Rboh isozymes present in this non-climacteric fruit. This study therefore focuses on providing a basic biochemical characterization of Rboh activity in sweet pepper fruits and its profile during the ripening process.

## 2. Materials and Methods

### 2.1. Plant Material 

**Figure 1:**
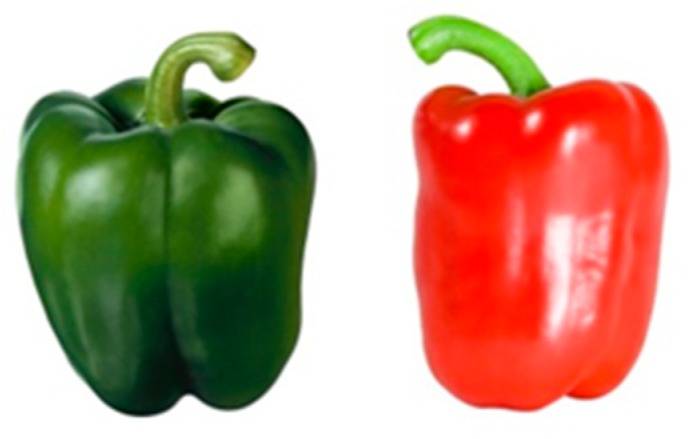
Sweet pepper (*Capsicum annuum* L.) fruit at distinct maturation stages (fully green and fully red).

California-type sweet pepper (*Capsicum annuum* L., cv. Melchor) fruits, obtained from Syngenta Seeds Ltd. (El Ejido, Almería, Spain), were used at two different ripening stages: Green immature and ripe red. Pepper plants were cultivated with optimal nutrient levels applied on rockwood as the soil substrate in the experimental greenhouse owned by Syngenta Seeds, Ltd., according to the usual crop program designed by the company, which usually involves planting seeds and germination in July to August, flowering in late September, and pollination and fruit setting from mid-October to late November [22]. Fresh fruits from the same plants at two distinct maturation stages (fully green and fully red) were collected at the same time from five different plants. Figure 1 shows a representative picture of the used sweet pepper fruits at the green and red ripening stages showing no external damages.

### 2.2. Preparation of Pepper Fruit Samples

Pepper fruits were ground in liquid N_2_ using a mortar and pestle, and the resulting powder was suspended in 50 mM Tris-HCl buffer, pH 7.5, containing 0.1 mM EDTA, 0.1% (*v*/*v*) Triton X-100, 1 mM MgCl_2_, 10% (*v*/*v*) glycerol to a final plant material/buffer ratio of 1:1 (*w*/*v*). Homogenates were then filtered through two layers of Miracloth and centrifuged at 27,000 g for 30 min. The supernatants were used for the assays.

### 2.3. Lipid Peroxidation Content

Lipid peroxidation was estimated by determining the thiobarbituric acid reacting substance (TBARS) content with the aid of malondialdehyde (MDA), which was used to prepare the standard curve [26].

### 2.4. Rboh Spectrophotometry Activity Assay

Superoxide (O_2_•^−^) generated by Rboh activity was measured using nitro blue tetrazolium (NBT) dye as an electron acceptor as described previously [27,28]. Briefly, NBT was rapidly converted to monoformazan by two O_2_•^−^ molecules. This reduction was spectrophotometrically detected at 530 nm. Monoformazan concentrations (thus equimolar to those of O_2_•^−^) were calculated using a 12.8 mM^−l^ cm^−l^ extinction coefficient. The NBT reduction rate was linear over time up to 15 min and linearly dependent on the protein concentrations in the pepper samples. The reaction contained a mixture of 50 mM Tris-HCl buffer (pH 7.8), 1 mM CaCl_2_, 0.1 mM NBT, 0.1 mM NADPH plus the sample in a final volume of 1 ml. For this spectrophotometric assay of Rboh activity in pepper fruit samples, it was crucial to eliminate low molecular weight compounds which could cause significant interference through a nonspecific reduction of NBT. The pepper samples were then loaded on a PD-10 desalting column containing Sephadex™ G-25 which enabled high (Mr > 5000) and low (Mr < 1000) molecular weight substances to be separated through desalting and buffer exchange, thus eliminating interference. No reduction in NBT in the absence of NADPH was observed in the pepper samples. As an additional control to evaluate the specificity of this assay, activity was also assayed in the presence of 50 µM diphenyleneiodonium (DPI) which specifically inhibits superoxide radical generation by Rboh [29]. 

### 2.5. In-Gel Rboh Activity Assay and Isozyme Profile

Rboh isozymes were separated using non-denaturing polyacrylamide gel electrophoresis (PAGE) on 6% acrylamide gels [30] and visualized by a photochemical NBT reduction method described by López-Huertas et al. [31] and modified by Sagi and Fluhr [2]. After the electrophoresis, the gels were briefly incubated in the dark for 20 min in a reaction mixture solution containing 50 mM Tris-HCl buffer (pH 7.4), 0.2 mM NBT, 0.1 mM MgCl_2_, and 1 mM CaCl_2_. Subsequently, 0.2 mM NADPH was added, and the appearance of the blue formazan bands was monitored. The reaction was stopped by immersing the gels in distilled water. As controls, gels were pre-incubated 30 min with 50 mM DPI, a specific inhibitor of superoxide radical generation by Rboh [29].

### 2.6. In Vitro Treatment with Nitric Oxide (NO) Donors, Peroxynitrite (ONOO^−^) and Glutathione

For the in vitro assay, red pepper samples were incubated at 25 °C for 1 h with increasing concentrations of different potential modulators, including diethylamine NONOate (DEA NoNoate) and *S*-nitrosocysteine (CysNO) as NO donors; L-cysteine (L-Cys) and reduced glutathione (GSH) [20,32,33]. In the case of the peroxynitrite donor SIN-1 (3-morpholinosydnonimine), the samples were incubated at 37 °C for 1 h [34,35]. In all cases, the solutions were freshly made before use. As an internal control, we determined that none of these chemicals oxidized NADPH in the reaction medium in the absence of the pepper samples.

### 2.7. Other Assays

Protein concentration was determined using the Bio-Rad protein assay (Hercules, CA), with bovine serum albumin as standard. Band intensity was quantified using ImageJ 1.45 software (https://imagej.nih.gov/ij/). Data are presented as the mean ± SEM of at least three independent biological replicates. With the aid of Statgraphics Centurion software, we used pairwise analysis of variance (ANOVA) to detect differences between green and red peppers.

## 3. Results

**Figure 2:**
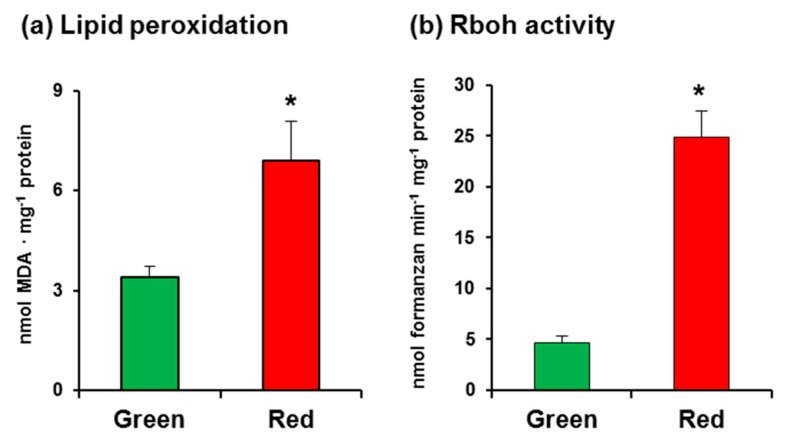
(**a**) Lipid peroxidation in pepper fruits. (**b**) Total respiratory burst oxidase homolog (Rboh) activity in pepper fruits. Data are the mean ± SEM of at least three independent biological replicates. Asterisks indicate that differences between values were statistically significant at *p* < 0.05.

In this study, we used California sweet pepper (*Capsicum annuum* L., cv. Melchor) fruits at the green and red ripening stages with no external damage (Figure 1). Malondialdehyde (MDA) content, which was measured as an index of lipid peroxidation, was observed to increase twofold in red fruits (Figure 2a), indicating that the ripening process involves oxidative stress. As part of an initial assay set-up for O_2_•^−^-generating Rboh activity in pepper samples, several controls were carried out, which showed that the crude extract samples have certain capacity to reduce NBT in the assay without the addition of NADPH (data not shown). Thus, to eliminate this interference, it was crucial to use PD-10 desalting columns containing Sephadex™ G-25 as indicated in the Materials and Methods section. This gel filtration step enabled us to obtain the protein fraction and to totally eliminate the nonspecific reduction of NBT. According to our assay, the activity in CaRboh was 53% higher in red fruits than in green fruits (Figure 2b).

**Figure 3:**
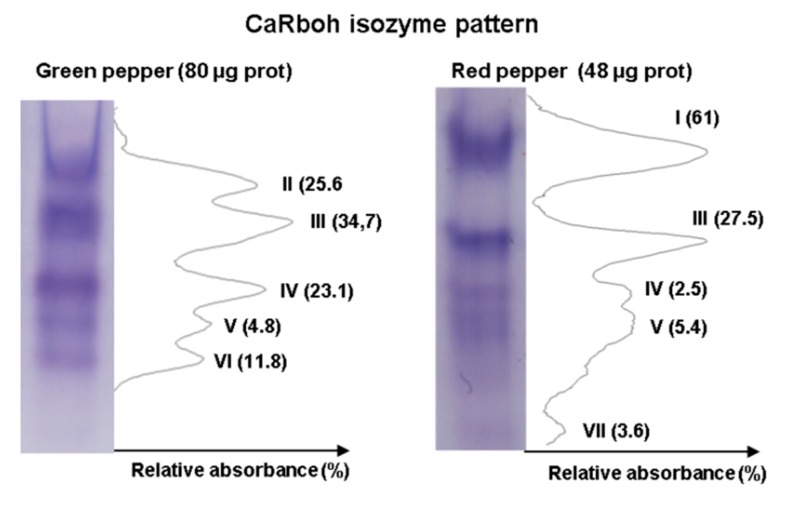
Activity of CaRboh isozymes in green and red pepper fruits. Protein samples were separated by non-denaturing polyacrylamide gel electrophoresis (PAGE, 6%) and activity was detected by the nitro blue tetrazolium (NBT) reducing method. For green and red peppers, 80 μg and 48 μg of protein were loaded per lane, respectively. Band intensity was quantified using ImageJ 1.45 software.

An additional step in the biochemical characterization of CaRboh activity is the detection of different isozymes by non-denaturing PAGE. Given that total CaRboh activity was higher in red fruits, total protein loaded per lane for each sample needed to be optimized in order to obtain clearly visible, well separated activity bands in the gels, as some activity bands could not be detected with low protein amounts, while the bands appeared too wide and overlapped when larger amounts of protein were used. Figure 3 shows the optimized CaRboh isozyme pattern in green fruits using 80 µg protein (Figure 3 left panel), with 48 µg protein being sufficient to detect well defined CaRboh isozymes in red fruits (Figure 3 right panel). Thus, a total of seven CaRboh isoforms, with different electrophoretic mobility and abundance, could be globally detected in green and red fruits as considered both together. Table 1 shows the number of each CaRboh isozyme (I to VII according to their increasing electrophoretic mobility) and its relative abundance in green and red fruits, with CaRboh I being the most prominent in red fruits, accounting for 61% of the total activity. In green fruits CaRbohIII was the most abundant isozyme with around 35% of the total activity. 

**Figure 4:**
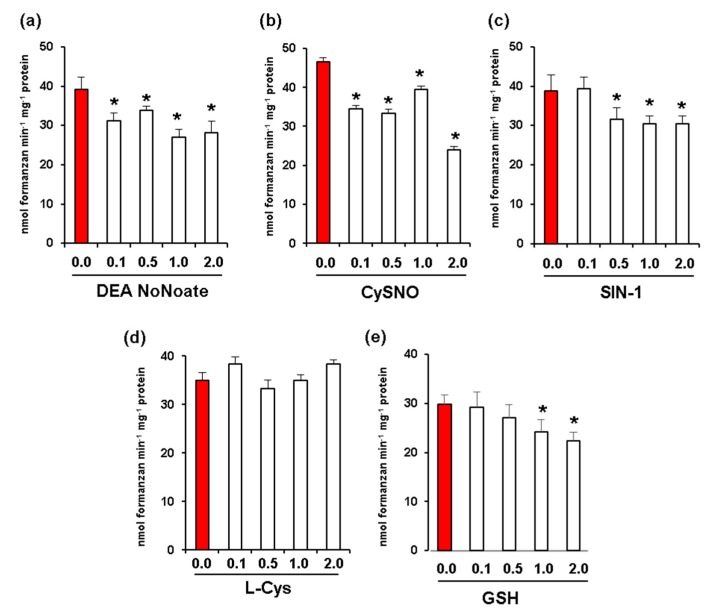
Effect of nitric oxide (NO) and peroxynitrite (ONOO^-^) on CaRboh activity in red green pepper fruits. (**a**) Effect of diethylamine NONOate (DEA NoNoate, nitric oxide donor). (**b**) Effect of S-nitrosocysteine (CySNO, NO donor). (**c**) Effect of 3-morpholinosydnonimine (SIN-1, peroxynitrite donor). (**d**) Effect of L-cisteine (L-Cys). (**e**) Effect of glutathione (GSH). Treatments with DETA-NONOate and *S*-nitrosocysteine (CySNO) as NO donors, L-Cys and GSH were done by incubating the pepper samples with these compounds at 25 °C for 1h. In the case of SIN-1, the assay was done at 37 °C for 1 h. Data represent the mean ± SEM of at least three independent biological replicates. Asterisks (*) indicate significant differences (*p* < 0.05) in comparison to the control.

Given that fruit ripening is associated with nitro-oxidative stress and changes in NO and *S*-nitrosothiol content [17,23], we carried out in vitro assays to analyze the potential effect of increasing concentrations of different NO donors including DEA NONOate and CysNO. In all these assays, we used red fruit samples due to their higher levels of Rboh activity. DEA NoNoate at a concentration of 2 mM was found to significantly inhibit Rboh activity by around 30% (Figure 4a), while CysNO caused an inhibition of 48% at the same concentration (Figure 4b). In addition, peroxynitrite (ONOO^−^) at a concentration of 2 mM resulted in an inhibition of 21% (Figure 4c). On the other hand, while L-Cys (Figure 4d) had no significant effect on Rboh activity at any of the concentrations assayed, 2 mM of reduced glutathione (GSH) promoted an inhibition of 25% (Figure 4e).

## 4. Discussion

Recently, the metabolism of ROS and RNS, which causes nitro-oxidative stress, was found to be active during sweet pepper ripening [36]. However, to our knowledge, no information is available on the potential involvement of Rboh activity in this process. In this study, we therefore analyzed Rboh activity during pepper ripening and identified the principal isozymes present in both green and red pepper fruits. 

### 4.1. Rboh Activity Increases during Pepper Fruit Ripening

Under our experimental conditions, the data obtained clearly show that total CaRboh activity significantly increases during pepper ripening, which opens up new questions about its potential role as the principal O_2_•^−^-generating enzyme [2]. This increase closely correlates with that observed in lipid peroxidation, a recognized marker of oxidative stress. However, its precise role is probably related to the ripening process, in which many biochemical pathways are redefined. In this respect, anthocyanin synthesis in the peels of apple (*Malus domestica*) fruits has been shown to directly correlate with an increase in Rboh activity [37], thus suggesting that O_2_•^−^ acts as a regulator of anthocyanin content. Anthocyanins are also well known to play an important role in determining the color of many fruits including some pepper fruit varieties [38,39].

On the other hand, it is well known that many fruits are stored at low temperature because this is a very effective method to extend the shelf life of fruits. However, there is experimental evidence which showed the correlation between cold induced oxidative injuries and the O_2_•^−^ generation by Rboh activity. Apple (*Malus domestica* Borkh) fruits stored for long periods at low temperatures can develop superficial injuries. The development of these damages is associated with oxidative reactions due to a burst of O_2_•^−^ and H_2_O_2_ as a consequence of Rboh activity, which leads to lipid peroxidation, cell membrane damage, and cell death [40,41]. Conversely, subtropical fruits such as mango or banana are sensitive to cold. For example, in mango (*Mangifera indica*) fruits storage at 5 °C it was observed an increase in lipid peroxidation with concomitant rises of genes involved in the linolenic acid oxidative pathway as well as of five *Rboh* genes [42]. Instead, this Rboh activity could have also some potential beneficial effects due to a transitory oxidative stress which stimulates the antioxidant system as a priming effect. Thus, mature green banana (*Musa acuminate*) fruits exposed to heat treatment previous to storage at 7 °C showed less cold damage. The reason is that this heat treatment triggered an increase in the expression of a *Rboh* gene which was accompanied by a concomitant increase of ROS content (O_2_•^−^ and H_2_O_2_) and ascorbate peroxidase (APX) activity/gene expression. Thus, authors suggested that the observed chilling resistance of banana fruits was correlated with the increase of the antioxidant system, specifically APX [43].

### 4.2. Total Rboh Activity is Inhibited in the Presence of NO Donors, Peroxynitrite and GSH

Given that NO metabolism is also modulated during pepper ripening when NO content diminishes [20,23,24,25], we used in vitro assays to analyze the inhibitory effect of NO and ONOO^−^ on CaRboh activity. Our findings are in line with those regarding *Arabidopsis thaliana*, in which the isoenzyme AtRboh D involved in plant immunity undergoes *S*-nitrosation at Cys890, leading to low O_2_•^−^ generation [44]. Similarly, in animal NADPH oxidase (NOX), *S*-nitrosation inhibits subunits of NOX2 [45] and NOX5 [46] isozymes. It has been well established that O_2_•^−^ reacts very rapidly with NO to generate ONOO^−^ (maximum rate constant for rapid reactions of around 4 × 10^9^ M^−1^ s^−1^) [47], which depends on the diffusion capacity of these two molecules. The potent nitrating molecule peroxynitrate can mediate protein Tyr-nitration [48] which reflects an active nitro-oxidative metabolism [49]. In this respect, the in vitro assay of ONOO^−^ showed that Rboh activity is inhibited in pepper samples, which suggests that nitration also takes place. To our knowledge, no information exists on this process occurring in NOX activity, which could be a cellular mechanism to limit the increase in O_2_^·-^ when ONOO^−^ content is already high. In pepper fruits, other enzymes, such as catalase and NADP-isocitrate dehydrogenase, which have been reported to be involved in controlling H_2_O_2_ and generating NADPH, are also inhibited by Tyr-nitration [20,23].

Glutathione (GSH), one of the most abundant antioxidants in plants, is also a component of cellular redox status. In previous reports using several varieties of pepper fruits it was reported that GSH decreased around 1.5-fold to 1.8-fold during ripening whereas ascorbate (ASC) content was unaffected [17,18]. In this context, it should be mentioned that GSH can interact with NO to generate *S*-nitrosoglutathione (GSNO), a physiological NO donor, which cellular content is regulated by the enzyme *S*-nitroglutathione reductase (GSNOR) that catalyzes the NADH dependent reduction of GSNO to oxidized glutathione (GSSG) and NH_3_ [50]. In sweet pepper fruits, it has been found that GSNOR activity and protein expression diminished during ripening whereas *S*-nitrosylated protein content increased [25]. Considering the close relationship between NO and GSH, it was also analyzed the potential effect of GSH. Under our experimental conditions, GSH was also observed to inhibit Rboh activity, thus suggesting the presence of a glutathionylation mechanism, which, to our knowledge, has never previously been reported in plant Rboh activity. However, a recent study shows that *S*-glutathionylation of NOX2 in human neutrophils allows O_2_^·−^generation to be maintained [51].

### 4.3. Isozymatic CaRboh Activity is Differentially Regulated in Green and Red Sweet Pepper Fruit 

In plants, Rbohs are encoded by a multigene family. For example, in the model plant *Arabidopsis thaliana*, up to ten *Rboh* genes (from *AtRboh* A to *AtRboh* J), which are differentially expressed depending on the tissue, organ, developmental stage, and environmental conditions, have been identified [52,53,54,55,56]. Similarly, up to nine *OsRbohs* in rice (*Oryza sativa*) and eight *SlRbohs* in tomato (*Solanum lycopersicum*) have been identified [57,58,59]. These Rbohs play a versatile role in plant reproduction, growth, development, and responses to abiotic and biotic stress [60,61]. Accordingly, the seven differentially expressed CaRboh isozymes identified in pepper fruits would appear to have adaptable functions, with CaRboh-III and CaRboh-I being the most prominent enzymes in green and red fruits, respectively. However, in a previous study of pepper leaves exposed to low temperature, only four Rboh isozymes were found [6]. Similarly, in strawberry (*Fragaria vesca*) plants, seven genes, with tissue-specific *Rboh* gene expression, have been identified: *FvRbohA*, *FvRbohC*, *FvRbohD*, and *FvRbohF* were detected in roots, stems, leaves, flowers, and fruits; *FvRbohB* and *FvRbohE* were expressed in roots, stems, flowers, and fruits; and *FvRbohH* was only present in flowers and fruits [10].

## 5. Conclusions

**Figure 5:**
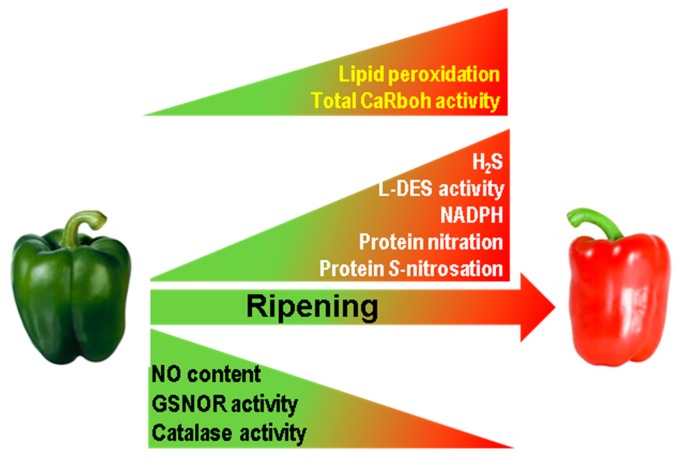
Reactive oxygen species (ROS) and reactive nitrogen species (RNS) metabolism during sweet pepper (*Capsicum annuum* L.) fruit ripening. In the model previously reported [20,36] it has been incorporated the total superoxide-generating CaRboh activity as well as the content of lipid peroxidation both increasing in red ripe fruits as reported in this work. GSNOR, nitrosoglutathione reductase. H_2_S, hydrogen sulfide. L-DES, L-cysteine desulfidrase.

This study, which provides novel insights into pepper fruit ripening, shows that superoxide-generating CaRboh activity increases during this process which closely correlates with the increase in lipid peroxidation and consequently with the physiological oxidative stress associated with pepper fruit ripening. Moreover, the number and abundance of the CaRboh isozymes identified are differentially regulated, suggesting some enzymatic specialization, with CaRboh-III and CaRboh-I being the most prominent isozymes in green and red fruits, respectively. All this opens up new lines of research to identify the specific functions of these isozymes in the ripening process. In addition, CaRboh activity appears to be regulated and inhibited by NO post-translational modifications, especially *S*-nitrosation, Tyr-nitration and probably also by glutathionylation. Taken together, these data suggest the connection between ROS and RNS metabolism during the physiological process of pepper fruit ripening. Figure 5 summarizes available information on how the different ROS and RNS parameters are modulated during pepper fruit ripening [20,36], incorporating data on Rboh activity and lipid peroxidation, and outlining the active nitro-oxidative metabolism in sweet pepper fruits. 

In this context, future research should be focused in the identification of specify enzymatic systems responsible of the NADPH generation during the fruit ripening necessary for the Rboh activity. Furthermore, NADPH is also required by the antioxidant system, specifically to regenerate the soluble antioxidant GSH by the enzyme glutathione reductase (GR) which is part of the ascorbate-glutathione cycle [61]. An illustrative example which shows the interconnection among all these elements (Rboh, NADPH, GSH, ascorbate, and antioxidant enzymes) has been described in a recent report on apple fruit. Thus, during postharvest store of this fruit it has been shown the relevance of Rboh and antioxidant activities which were supported by the NADPH-generating enzyme, glucose-6-phosphate dehydrogenase (G6PDH) allowing together an enhance disease resistance against blue mold [62].

## Figures and Tables

**Table 1:** Identification (I to VII) and relative abundance (%) of the respiratory burst oxidase homolog (CaRboh) isozymes detected in green and red pepper (*Capsicum annuum* L.) fruits by the NBT reducing method after non-denaturing PAGE. Band intensity was quantified using ImageJ 1.45 software.

Pepper Fruit Stage	CaRboh Isozymes (%)						
	I	II	III	IV	V	VI	VII
Green	ND	25.6	34.7	23.1	4.8	11.8	ND
Red	61	ND	27.5	2.5	5.4	ND	3.6

ND: not detected.
